# Iron-Modified Biochar Strengthens Simazine Adsorption and Decreases Simazine Decomposition in the Soil

**DOI:** 10.3389/fmicb.2022.901658

**Published:** 2022-07-01

**Authors:** Hongguang Cheng, Dan Xing, Shan Lin, Zhaoxia Deng, Xi Wang, Wenjing Ning, Paul W. Hill, David R. Chadwick, Davey L. Jones

**Affiliations:** ^1^State Key Laboratory of Environmental Geochemistry, Institute of Geochemistry, Chinese Academy of Sciences, Guiyang, China; ^2^School of Natural Science, Bangor University, Bangor, United Kingdom; ^3^Institute of Pepper Guiyang, Guizhou Academy of Agricultural Science, Guiyang, China; ^4^Key Laboratory of Arable Land Conservation (Middle and Lower Reaches of Yangtze River), College of Resources and Environment, Huazhong Agricultural University, Ministry of Agriculture, Wuhan, China; ^5^College of Resources and Environment Engineering, Guizhou University, Guiyang, China; ^6^College of Resources and Environment, Yangtze University, Wuhan, China; ^7^SoilsWest, Centre for Sustainable Farming Systems, Food Futures Institute, Murdoch University, Murdoch, WA, Australia

**Keywords:** iron-modified biochar, simazine, decomposition, adsorption, microbial community

## Abstract

Currently, modified biochar has been successfully used in the remediation of soil polluted with heavy metals. However, the effects of the modified biochar on pesticides (such as simazine) are still unclear. Herein, the environmental fate of simazine, such as decomposition, leaching, and adsorption in unamended soil, in the soil amended with unmodified and modified biochar (biochar + FeCl_3_, biochar + FeOS, biochar + Fe) were evaluated. In addition, an incubation experiment was also performed to observe the influence of modified biochar on the microbial community and diversity in the soil. The results showed that modified biochar significantly decreased the decomposition of simazine in the soil compared to its counterpart. Modified biochar also reduced the concentration of simazine in the leachate. Compared with the control, soil microbial biomass in the soil amended with unmodified biochar, biochar + FeCl_3_, biochar + Fe, and biochar + FeOS was decreased by 5.3%, 18.8%, 8.7%, and 18.1%, respectively. Furthermore, modified biochar changed the structure of the microbial community. This shows that modified biochar could increase the soil adsorption capacity for simazine and change the amount and microbial community that regulates the fate of simazine in the soil. This study concludes that iron-modified biochar has positive and negative effects on the soil. Therefore, its advantages and side effects should be considered before applying it to the soil.

## Introduction

As the second most detectable pesticide in surface and groundwater in the United States, Europe, and Australia, simazine can be found in water at hundreds of micrograms per liter (Cox et al., 2000; Troiano et al., 2001). This has raised many environmental issues, including human health, aquatic, and terrestrial ecosystems (Regitano et al., 2006; Rico et al., 2012). In order to prevent ground and surface waters from being contaminated with simazine, strategies were implemented to cut down on the persistence of simazine in the environment. For example, organic matter content as a modifier was applied to the soil to regulate herbicide behavior (Cox et al., 2001; Odukkathil and Vasudevan, 2013). Multiple technologies that can be used to control pesticides in the soil, including physiochemical technologies and biological methods, have shortcomings such as low efficiency, long time, high cost, and even the introduction of new pollution, which result in several problems in the remediation of pesticide pollution (Fan and Song, 2014; Meriam Suhaimy et al., 2020) that have not been solved. In recent years, biochar application as an amendment for remediation of soil and water pesticide pollution has attracted increasing attention worldwide. Several studies indicated that biochar could effectively increase the adsorption of simazine in the soil, thus decreasing its risk of leaching into the environment and also reducing its risk of being directly being up-taken by plants (Jones et al., 2011a; Eibisch et al., 2015; Cheng et al., 2021). Simultaneously, some studies have reported that biochar inhibits simazine biodegradation (Cheng et al., 2017a) and reduces simazine efficacy for controlling weeds or killing pests. The studies mentioned above also show that the application of biochar can regulate the transport and decomposition of pesticides in the soil, thereby avoiding soil and water pollution.

Due to its high surface area and stronger cation exchange capacity, biochar can be used as a modifier to remediate soil contaminated by simazine (Zheng et al., 2010; He et al., 2019). When applied to the soil, the increased surface area and cation exchange capacity increase the soil adsorption capacity for simazine (Cheng et al., 2017a; Liu et al., 2018), resulting in lower pesticide concentrations in leachate (Larsbo et al., 2013; Tatarková et al., 2013) and crop residues (Yang et al., 2010). Additionally, when more simazine is adsorbed by the soil, the probability of soil microorganisms coming into contact with simazine is reduced, which reduces the rate of simazine decomposition (Cheng et al., 2017a). This also increases the risk of accumulating simazine in the surface soil. Therefore, biochar addition is a risk-benefit approach for simazine pollution control in agricultural soils. To take things a step further in enhancing the improvement effect, much-modified biochar is applied to agricultural soils to improve the soil adsorption capacity. However, this method can reduce the contact chance of simazine with soil microorganisms and may also reduce the number of microorganisms in the soil, thereby inhibiting the decomposition of simazine.

In recent years, biochar modified with iron materials has been widely used to control heavy metal (especially arsenic and cadmium) pollution in the agricultural soil (Wu et al., 2018, 2019; Pan et al., 2019). The addition of iron-based materials enhanced the specific surface area of biochar and improved its reactivity (Asmel et al., 2017; Peng et al., 2021). Simultaneously, iron can increase the number of specific adsorption sites and change the chemical activity of the adsorbent by changing the pH value (at the point of zero charges), ultimately promoting electrostatic interactions between heavy metals and the adsorbent surface (Vieira et al., 2017). This greatly improved the adsorption performance of biochar for heavy metals (Cui et al., 2021; Yuan et al., 2021). Inevitably, the above process also increases the adsorption of simazine by biochar and may even affect the composition and quantity of the soil microbial community, thereby changing the turnover of simazine in the soil. Although the remediation of iron-modified biochar on heavy metals has received significant attention, studies on their effects on simazine fate are rare, especially the influence of microbes regulated by iron-modified biochar on simazine decomposition. Therefore, this study was aimed at (1) comparing simazine fate, including decomposition, adsorption, and leaching in the soil amended with iron-modified biochar. It was also aimed at (2) investigating the possible mechanisms for simazine fate by iron-modified biochar and (3) evaluating the influence of iron-modified biochar application on the fate of simazine in soil.

## Materials and Methods

### Biochar Preparation, Soil, and ^14^C-Labeled Simazine

Unmodified biochar was produced from wheat (Triticum aestivum L.) straw collected from the Henfaes Research Centre Wales, North Wales, United Kingdom (53° 140N; 4° 100W). The preparation process was introduced by Cheng et al. (2021). The iron modification process was mainly completed according to the method developed by Wu et al. (2018). Briefly, biochar was first roasted and then impregnated with a mixture solution (100 ml, 0.75 mol/L) of FeSO_4_⋅7H_2_O and FeCl_3_ solution (100 ml, 0.75 mol/L), in which the n (FeSO_4_⋅7H_2_O): n (H_2_O_2_) ratio was set at 1:0.5. After that, the solution was stirred and filtered, followed by drying the unfiltered materials at room temperature. These materials were collected for subsequent use and named biochar-FeOS and biochar-FeCl_3_. In addition, in this study, biochar was thoroughly mixed with zero-valent iron under a continuous flow of nitrogen gas (N_2_), called biochar-Fe.

Soil was collected from the field in the Henfaes Research Centre, which is used for grassland and arable production. Soil (Eutric Cambsiol soil type) in the Ah horizon (0–15 cm, sandy loam) was collected in bags and transported to the laboratory. It was naturally dried and sieved through a 2 mm mesh sieve to remove plant residues and stones. The major properties of the soil are shown in Table 1, with additional properties displayed by Farrar et al. (2012) and Jones et al. (2012).

**TABLE 1:** The physical and chemical characterization of soil.

pH	Ec (uScm^–1^)	TC (%)	TN (%)	TOC (mg/kg)	NO3^–^ (mg/kg)	NH4^+^ (mg/kg)
5.20 ± 0.02	87.73 ± 4.18	3.10 ± 0.05	0.34 ± 0.01	411.96 ± 25.57	4.13 ± 0.23	12.8 ± 0.20

Simazine (6-chloro-N, N’-diethyl-1,3,5-triazine-2,4-diamine) was purchased from Sigma Chemical Co (St. Louis, MO, United States). Before the experiment, simazine was labeled with a simazine ^14^C stock solution.

### Experiment Design

In this study, five treatments were installed: (1) Soil without biochar (CK); (2) a mixture of unmodified biochar and soil at a ratio of 1:25 (BC); (3) a mixture of modified biochar (biochar-FeOS) and soil at a ratio of 1:25 (BC-FeOS); (4) a mixture modified biochar (biochar- FeCl_3_) and soil at a ratio of 1:25 (BC-FeCl_3_); (5) a mixture of modified biochar (biochar-Fe) and soil at a ratio of 1:25 (BC-Fe).

#### Decomposition Experiment

Approximately 300 g of the prepared soil was packed into a PV box (L × W × H = 11 × 8 × 10.5 cm), and the humidity was adjusted to 70% of the water holding content (Jones et al., 2011b). These samples were stored at 20°C for 14 days for microbial recovery. Then 5.0g of soil was transferred into a 50ml centrifuge tube. Then, 0.5 ml of ^14^C-labeled simazine (0.60 mg L^–1^, 0.54 kBq mL^–1^) was added to each tube. A 1-ml NaOH trap (1 M) was placed above the sample to capture CO_2_ released from the sample. The NaOH traps were replaced on the 1^st^, 3^rd^, 5^th^, 7^th^, 10^th^, 14^th^, 17^th^, and 21^st^ days. Finally, the tubes were sealed and incubated in the dark at 20°C for 21 days. The ^14^CO_2_ content in the NaOH traps was determined by liquid scintillation counting using Optiphase 3 scintillation fluid (PerkinElmer Corp., Waltham, MA) and a Wallac 1404 liquid scintillation counter (PerkinElmer Corp). In addition, 10.0 g of other soil was collected and analyzed using phospholipid fatty acids (PLFA).

#### Adsorption Experiment

A series of batch experiments were performed to obtain the sorption isotherms of simazine by soil amended with or without biochar and iron-modified biochar. First, 2.0 g previously prepared soil sample was weighed into a 50 ml centrifuge tube. Before adding liquid, these loaded soil tubes were put into the oven at 80°C for 30 min to minimize microbial degradation (Kuzyakov and Jones, 2006). Then a 20ml solution containing ^14^C-labeled simazine (0.05 Kbq ml^–1^) was added to each tube. To balance the salt ionic, CaCl_2_ was added to the tube at a concentration of 0.01 mol L^–1^ to balance the salt ionic. The concentration of simazine in the solution ranged from 0, 6.25, 12.5, 25.0, 50.0, and 100.0 μg/L. After that, the samples were shaken at 200 rpm for 24 h at 20°C. Then 1 ml of the supernatant was extracted from the soil solution and subjected to centrifugation at 4000 rpm for 10 min to determine ^14^C activity. The ^14^C activity measurement was the same as in the above introduction.

#### Leaching Experiment

The details of the leaching experiment were introduced by Jones et al. (2011a) and Cheng et al. (2017b). Briefly, 5 g of the previous soil sample was weighed into a 25 ml inverted syringe (2 cm in diameter). A 1 mm polypropylene mesh was placed at the bottom, and the other was placed on the soil’s surface to prevent soil loss and solution shock. Then 1 ml of ^14^C-labeled simazine (2.50 mg L^–1^, 0.05 kBq ml^–1^) was added to the soil surface. After that, a syringe pump was used to add distilled water at 0.2 ml/min after waiting for 1 h of equilibration period. The resulting leachate was sequentially collected (equivalent to 1, 2, 3, 4, 5, and 6 soil pore volumes), and its ^14^C activity was determined as described above.

### Analysis of Microbial Communities

PLFA analysis was used to provide a general profile of the microbial community and quantify total microbial biomass because PLFA is the main component of all microbes’ cell membranes (Kim et al., 2018; Zang et al., 2020). Therefore, soil samples collected and stored at −80^°^C before adding simazine were undergone for PLFA analysis of microbial communities. PLFA was performed according to the method of Buyer and Sasser (2012) with different taxonomic groups classified as described in the study of Frostegård et al. (1993) with acknowledgment of the caveats raised by Frostegård et al. (2011). The soil was suspended in a solution of a methanol-chloroform-phosphate buffer. After filtration, the chloroform phase was separated, and the phospholipids were separated from glycolipids and neutral lipids by solid-phase extraction. The phospholipids were saponified and methylated to fatty acid methyl esters using an Agilent 6890 gas chromatograph equipped with a flame ionization detector and an Ultra-2 column (Kim et al., 2018).

### Physicochemical Properties Analysis

All analyses for collected samples were repeated four times. Two solutions were prepared, in which one solution was prepared by mixing a soil dry sample with deionized water (1:2.5, w:v). Another solution was prepared by mixing biochar and deionized water suspension with standard electrodes (1:2.5, w:v). Available NO_3_^–^ and NH_4_^+^ were measured using a colorimetric method (Mulvaney, 1996; Miranda et al., 2001) based on soil extractions (0.5 M K_2_SO_4_ extracted from biochar and soil). Soil organic carbon was measured using the K_2_Cr_2_O_7_ oxidation method. Ash content of the biochar was measured according to the weight loss of biochar when combusted at 575°C for 16 h. Elemental C, N, H, and S abundances were determined using a Vario MACRO cube analyzer. O content was calculated based on the assumption that biochar is composed of C, N, H, and O only after deducting the ash content. The biochar’s Cation exchange capacity (CEC) was determined according to the modified ammonium acetate method (Gaskin et al., 2008). WHC of the biochar was determined according to EBC (2012). The biochar’s specific surface area (SSA) was measured using an Autosorb iQ/monosorb surface area analyzer (Quantachrome Instruments, Boynton Beach, FL, United States). Functional groups were determined using a Fourier transform infrared spectrometer (FTIR). The zeta potential was determined using a Malvern Zeta meter (Nano ZSE + MPT2, Malvern Panalytical Instruments Ltd., United Kingdom). The surface morphology of the biochar was observed using scanning electron microscopy (SEM) JSM-6460 LV Scanning Microscope (JEOL, Tokyo, Japan).

### Statistical Analysis

As displayed in equation (1), the distribution coefficient (Kd) was calculated from the difference between the total amount added and the amount that stayed in the solution.


(1) Kd=(C0-Ce)×V/W/Ce

where W is the weight of the soil sample, V is the volume of CaCl_2_ including the simazine solution, C_0_ is the concentration in the starting solution, and Ce is the concentration of simazine in the solution after adsorption.

The adsorbed results were fitted by the Langmuir and Freundlich models. The Langmuir model is expressed as Equation (2), and the Freundlich isotherm model is described as Equation (3).


(2) $$\frac{C_{\text{e}}}{q_{\text{e}}}=\frac{1}{q_{\max}\,K_{\text{L}}}+\frac{C_{\text{e}}}{q_{\max}}$$

(3) $$\ln q_{\text{e}}={\mathrm{lnK}}_{\text{F}}+\frac{1}{\text{n}}\,\text{ln}\,C_{\text{e}}$$

where q_*e*_ and q_*max*_ are the equilibrium and maximum adsorption capacities (mg/g), respectively, KL is the Langmuir constant related to the affinity of the binding sites (L/mg), and Ce is the equilibrium concentration of adsorbate in an aqueous phase (mg/L). K_*f*_ is a constant that represents Freundlich adsorption capacity, and n is a constant that represents the adsorption intensity.

The variables, including biochar and soil properties and ^14^C activity in adsorption, decomposition, and leaching experiments among treatments, were first tested for normality and homogeneity of variance. Variables with normal distributions and equality of variance were analyzed using a one-way ANOVA with Fisher’s least significant difference (LSD). Variables with non-normal distributions or unequal variance (decomposition and leaching) were studied non-parametrically using a Wilcoxon paired signed-rank test. All differences were considered significant at the *p* < *0.05* level. Linear regression was undertaken in Origin 2019b.0 (OriginLab Corp, Northampton, MA).

## Results and Discussion

### Biochar and Iron-Modified Biochar Properties

**FIGURE 1:**
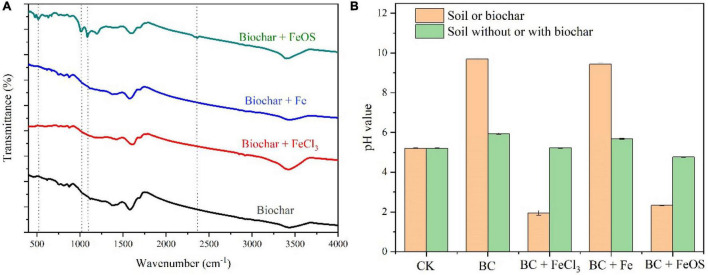
FTIR spectra for the pristine biochar and iron-modified biochar **(A)** and pH value of soil before and after biochar amendment **(B)**.

**FIGURE 2:**
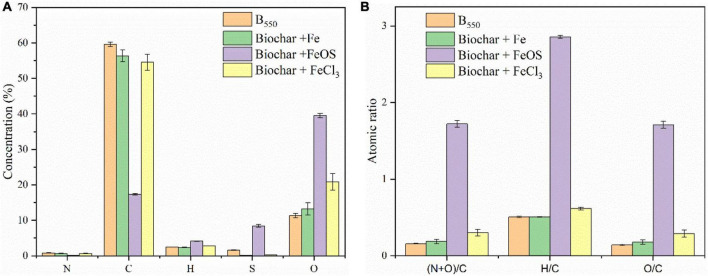
The content of elements **(A)** and the molar ratio **(B)** of biochar or iron modified biochar.


The chemical and physical properties of the iron-modified biochar are listed in Figure 1 and Table 1. Compared to BC (9.70), except for BC-Fe (9.44), the pH value was significantly lower in BC-FeCl_3_ (1.95) and BC-FeOS (2.33) after iron modification. C, H, N, and O content of biochar or iron-modified biochar are shown in Figure 1. The C content in BC was 59.59%, 56.34% in BC + Fe, and 54.57% in BC + FeCl_3_, whereas it was decreased to 17.35% in BC + FeOS. The O content of BC (11.33%) was significantly lower than BC + Fe (13.26%), BC + FeOS (39.53%) and BC + FeCl_3_ (20.82%). Generally, biochar prepared above 250 degrees will be alkaline (Cheng et al., 2017a), probably because of oxygen-containing functional groups and carbonate substances in biochar (Yuan et al., 2011). The biochar prepared at 550^°^C showed strong alkalinity (9.70). This is consistent with the previous reports (Mandal et al., 2018; Tomczyk et al., 2020). However, after modification with FeCl_3_ and FeOS, the pH of the modified biochar decreased significantly (Figure 1B and Table 1). Infrared spectroscopy results showed that the modified biochar’s oxygen-containing functional groups changed significantly compared to pristine biochar (Figure 1A). At the same time, the composition of carbon, oxygen, hydrogen, nitrogen, and other elements has undergone obvious changes during the modification process (Figure 2A). This suggests that the carbonate substances in the pristine biochar have undergone significant chemical reactions. Although the change of oxygen-containing functional groups and carbonate substances during the modification process led to a decrease in pH value for the modified biochar, the main reason was that the hydrolysis reaction of FeOS and FeCl_3_ occurred in (4) during the modification process, releasing a large amount of H ions that resulted in the strong acidity of BC-FeOS and BC-FeCl_3_ (Wu et al., 2018; Zhang et al., 2020).

(4) Fe+3+HO2=Fe(OH)+3H+

The atomic ratios of C, N, H, and O elements [H/C, O/C, and (N + O)/C] represent the aromaticity, hydrophilicity, and polarity of biochar (Wu et al., 2018). As evinced in Figure 2A, The carbon content of the pristine biochar was significantly reduced after the modification of FeOS, while the content of H, N, and O increased accordingly. The main reason is that the organic carbon and inorganic carbon undergo strong oxidation and neutralization reactions in the modification process of the pristine biochar, and CO_2_ is generated and released into the atmosphere, resulting in a significant decrease in the carbon content of the modified biochar, which coincides as the relative content of H, N, and O elements rise. In addition, the atomic ratios of H/C, O/C, and (N + O)/C were significantly higher in BC-FeOS than in pristine biochar or other iron-modified biochar, indicating that BC-FeOS has high aromaticity, hydrophilicity, and polarity (Figure 2B).

### The Influence of Iron Modified Biochar on Simazine Adsorption

**FIGURE 3:**
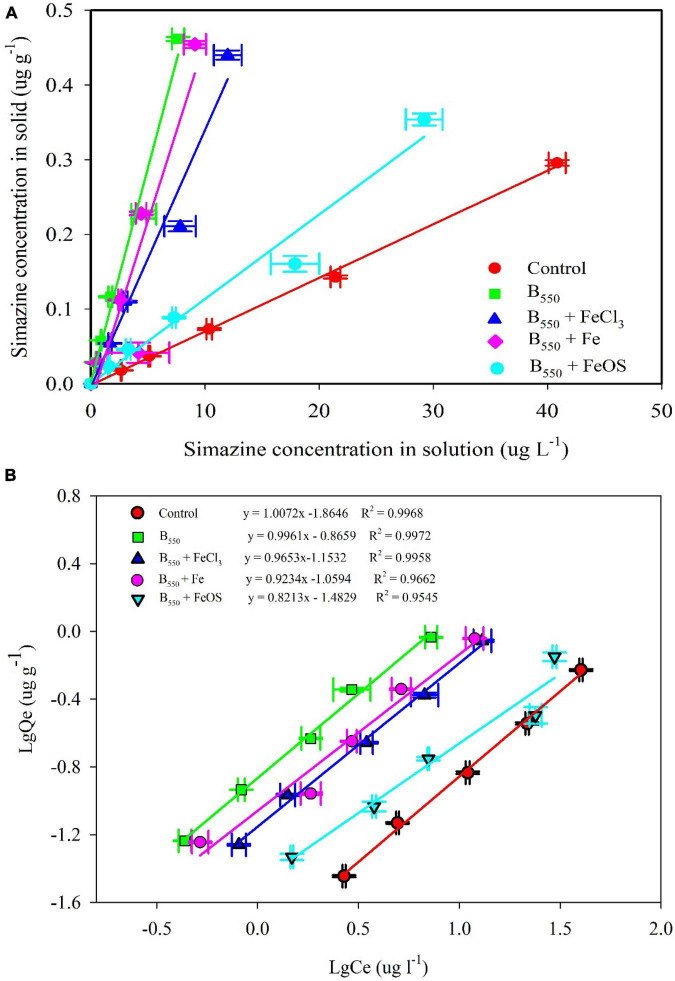
The adsorption of iron modified biochar or pristine biochar on simazine. Distribution of simazine in solid and liquid phases **(A)** and Freundlich isotherms **(B)**.

As shown in Figure 3, iron significantly changed the adsorption of simazine on biochar. Kd values decreased from 57.7 L kg^–1^ in pristine biochar to 11.3L kg^–1^ in BC + FeOS. Nonetheless, the addition of iron-modified biochar significantly increased the adsorption of simazine from the soil compared to the adsorption of simazine by the control soil (Kd 7.2 kg^–1^). In addition, according to the R^2^ value of the Langmuir and Freundlich models (*R*^2^ > 0.97, Table 2), the Freundlich models can better fit the adsorption of simazine on iron-modified biochar. The findings showed that adsorption capacity constants K_*f*_ were 0.014 in control, 0.136 in BC, 0.070 in BC-FeCl_3_, 0.087 in BC-Fe, and 0.033 in BC-FeOS, respectively. Evidently, those adsorption capacity constants indicated that the iron modification significantly decreased the adsorption capacity compared with the pristine biochar. This view is supported by the partitioning results of simazine between solid and liquid phases at adsorption equilibrium (Figure 3).

**TABLE 2:** The properties of biochar with and without iron modification.

	Biochar	Biochar-FeCl_3_	Biochar- FeOS	Biochar-Fe
pH	9.70 ± 0.10	1.95 ± 0.12	2.33 ± 0.01	9.44 ± 0.07
Ec (uScm^–1^)	4.56 ± 0.29	12.270.92	5.920.28	4.27 ± 0.92
CEC (cmol kg^–1^)	2.07 ± 0.16	2.14 ± 1.10	18.03 ± 0.65	2.03 ± 0.18
WHC (%)	659.77 ± 9.14	411.41 ± 5.09	97.20 ± 5.71	222.40 ± 5.67
Zeta potential (mv)	−31.87 ± 1.91	−24.76 ± 0.73	−17.42 ± 1.91	−33.24 ± 1.98

Previous studies have shown that the organic carbon content in biochar has an essential effect on the adsorption of simazine (Yang et al., 2016; Feng et al., 2021). In this study, a series of complex oxidation reactions occurred during iron modification (Wu et al., 2018), resulting in lots of organic carbon in the pristine biochar, which was oxidized and escaped. As a result, the adsorption capacity of iron-modified biochar to simazine was reduced. The element content in biochar (Figure 2A) also showed low carbon content in the iron-modified biochar, especially in BC-FeCl_3_ and BC-FeOS. In addition, the content of element C in biochar was inversely proportional to the adsorption capacity of biochar to simazine. The above phenomenon indicated that the carbon content in biochar greatly influences the adsorption capacity of biochar to simazine. However, iron modification reduced the carbon content in the modification process, which decreased the adsorption of biochar to simazine.

### The Influence of Iron Modified Biochar on Simazine Leaching

**FIGURE 4:**
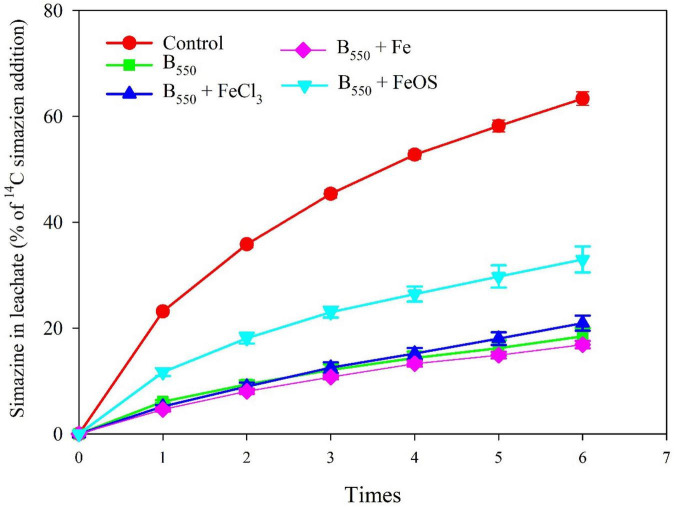
The concentration of simazine in the leachate.

The concentration of simazine in the leachate directly results from the performance of biochar in the adsorption process, which is associated with the adsorption capacity of biochar (Liu et al., 2018). In this study, 63.36% of simazine was leached out in the control leachate, which has the highest content among all treatments. However, the exudation of simazine was 18.43% in BC, 32.95% in BC + FeOS, 16.86% in BC + Fe and 20.92% in BC + FeCl_3_ (Figure 4). Apparently, the addition of biochar significantly reduced the concentration of simazine in the leachate. Moreover, the iron modification processes weakened the retention capacity of biochar for simazine, increasing groundwater pollution compared with pristine biochar. The desorption process is the reverse process of adsorption onto the adsorbent (Cabrera et al., 2014). The stronger the adsorption capacity of the adsorbent to the adsorbed substance, the less the adsorbed substance can be desorbed. The concentration of simazine in the leachate (Figure 3) and the adsorption of iron-biochar to simazine (Figure 2A) indicated that the content of simazine in the leachate was regulated by the adsorption capacity of biochar to simazine. Therefore, iron modification leads to a decrease in biochar adsorption capacity for simazine, which increases the potential risk of simazine migration into watercourses.

### Regulation of the Microbial Community and Simazine Decomposition

**FIGURE 5:**
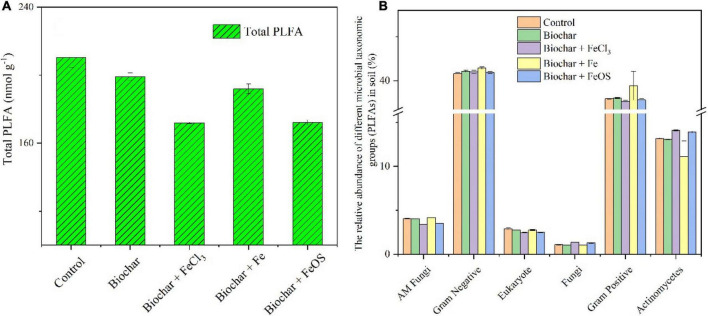
The influence of iron modified biochar on the total biomass **(A)** and the relative abundance of different microbial taxonomic groups **(B)** (PLFAs) in soil.

The application of biochar can improve the physical and chemical properties of the soil (Keith et al., 2016). It can also increase the ability of soil to retain water and fertilizer (Hariz et al., 2015). At the same time, it can directly provide nutrients, further affect the composition and activity of microorganisms in the soil (Yang et al., 2021), and regulate the amount of soil organic matter or exogenous organic matter decomposition (Cheng et al., 2017a). In addition, the addition of biochar can reduce the contact probability between exogenous organic matter and microbial decomposers, thereby reducing the decomposition of exogenous organic matter (Cheng et al., 2017b). The total microbial biomass and microbial community were also studied as shown in Figures 5A,B. After biochar application in the soil, the total microbial biomass decreased (Figure 5A). Compared to the control, the total microbial biomass in the soil with the addition of unmodified biochar decreased by 5%. The most significant effect was that the total microbial biomass in soils treated with BC + FeCl_3_ and BC + FeOS additions decreased by 18%. The possible reason was the change in soil’s physical and chemical properties due to biochar addition (Yang et al., 2021). According to the soil pH (Figure 2B), it is evidenced that soil acidification inhibits the growth of soil microorganisms (Figure 5A). In addition, the results in the composition of the community structure showed that the composition of *AM fungi* and *Eukaryote*s in the soil amended with BC + FeCl_3_ or BC + FeOS was significantly lower than that of the control treatment (Figure 5B).

**FIGURE 6:**
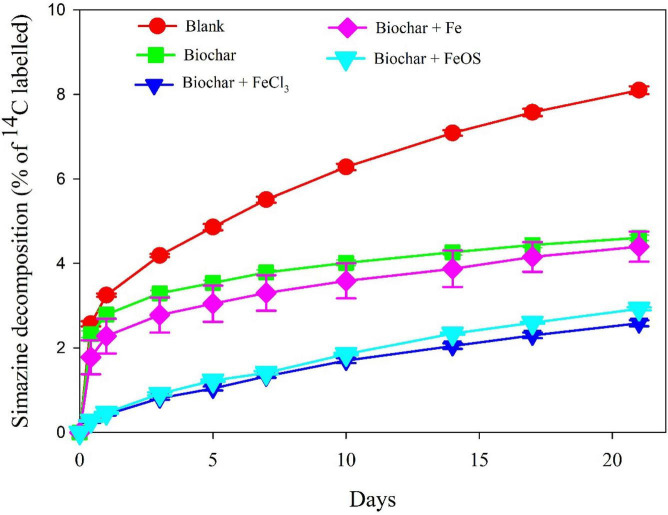
The simazine decomposition in the soil amended with biochar or iron modified biochar.

As shown in Figure 6, the decomposition of simazine in the soil was observed after iron-modified biochar was applied. As previously reported, biochar addition inhibited the decomposition of simazine in soil (Cheng et al., 2017b). Compared with the control, the decomposition ratio of simazine in the pristine biochar amended soil decreased by 43.13%, and the decomposition ratio of simazine in the BC + FeCl_3_ and BC + FeOS addition treatments decreased by 68.09% and 63.88%, respectively. Obviously, the iron-modified biochar showed a more substantial inhibitory effect on the decomposition of simazine. Previous studies considered that the addition of biochar to the soil enhanced the adsorption of pesticides, thereby reducing the probability of contact between pesticides and soil microbial decomposers and reducing the decomposition rate of pesticides in the soil. This shows that the adsorption of pesticides by soil after biochar addition could regulate the decomposition rate of pesticides (Liu et al., 2018). However, the adsorption of iron-modified biochar to simazine and the decomposition of simazine in iron-modified amended soil showed that the adsorption of simazine in soil and the inhibition of simazine decomposition by biochar did not completely correspond. On the contrary, the effect of biochar on the total microbial biomass and the change of community composition compared to the decomposition of simazine indicates that the impact of biochar on the composition and structure of the microbial community played a prominent role in the decomposition of simazine in soil.

## Conclusion

In summary, adding biochar modified with iron in the soil evinced a significant effect on simazine because it increased simazine adsorption and regulated microbial community content. Therefore, the adsorption of simazine in soil amended with iron-modified biochar was significantly higher. The decomposition of simazine in soil amended with iron-modified biochar was substantially lower. Compared to the control, the adsorption of simazine was increased in the treatment with biochar addition. However, compared to pristine biochar, iron modification decreased the adsorption of simazine. At the same time, iron-modified biochar addition to soil significantly reduced simazine decomposition. The comprehensive analysis has revealed the increased adsorption of simazine due to biochar addition, which decreased the probability of simazine exposure to microorganisms. The influence of iron-modified biochar on microbial biomass and the community was the main reason for simazine decomposition.

## Data Availability Statement

The raw data supporting the conclusions of this article will be made available by the authors, without undue reservation.

## Author Contributions

HC: conceptualization and writing and experiment. DX and SL: review and editing. ZD: data curation. XW and WN: experiment. PH, DC, and DJ: conceptualization and English improvement. All authors contributed to the article and approved the submitted version.

## Conflict of Interest

The authors declare that the research was conducted in the absence of any commercial or financial relationships that could be construed as a potential conflict of interest.

## Publisher’s Note

All claims expressed in this article are solely those of the authors and do not necessarily represent those of their affiliated organizations, or those of the publisher, the editors and the reviewers. Any product that may be evaluated in this article, or claim that may be made by its manufacturer, is not guaranteed or endorsed by the publisher.
